# CNP mediated selective toxicity on melanoma cells is accompanied by mitochondrial dysfunction

**DOI:** 10.1371/journal.pone.0227926

**Published:** 2020-01-17

**Authors:** Elif Aplak, Claudia von Montfort, Lisa Haasler, David Stucki, Bodo Steckel, Andreas S. Reichert, Wilhelm Stahl, Peter Brenneisen

**Affiliations:** 1 Institute of Biochemistry and Molecular Biology I, Medical Faculty, Heinrich Heine University Düsseldorf, Düsseldorf, Germany; 2 Department of Molecular Cardiology, Medical Faculty, Heinrich Heine University Düsseldorf, Düsseldorf, Germany; National Institutes of Health, UNITED STATES

## Abstract

Cerium (Ce) oxide nanoparticles (CNP; nanoceria) are reported to have cytotoxic effects on certain cancerous cell lines, while at the same concentration they show no cytotoxicity on normal (healthy) cells. Redox-active CNP exhibit both selective prooxidative as well as antioxidative properties. The former is proposed to be responsible for impairment of tumor growth and invasion and the latter for rescuing normal cells from reactive oxygen species (ROS)-induced damage. Here we address possible underlying mechanisms of prooxidative effects of CNP in a metastatic human melanoma cell line. Malignant melanoma is the most aggressive form of skin cancer, and once it becomes metastatic the prognosis is very poor. We have shown earlier that CNP selectively kill A375 melanoma cells by increasing intracellular ROS levels, whose basic amount is significantly higher than in the normal (healthy) counterpart, the melanocytes. Here we show that CNP initiate a mitochondrial increase of ROS levels accompanied by an increase in mitochondrial thiol oxidation. Furthermore, we observed CNP-induced changes in mitochondrial bioenergetics, dynamics, and cristae morphology demonstrating mitochondrial dysfunction which finally led to tumor cell death. CNP-induced cell death is abolished by administration of PEG-conjugated catalase. Overall, we propose that cerium oxide nanoparticles mediate cell death via hydrogen peroxide production linked to mitochondrial dysfunction.

## 1. Introduction

In recent years, nanomedicine has gained a lot of interest because of their possible biomedical application. Due to their mixed valence states of Ce^3+^ and Ce^4+^, cerium (Ce) oxide nanoparticles (CNP) are able to affect the redox homeostasis of cells [1]. Redox-based therapies show very promising results [1, 2], especially the SOD-mimetic as well as the catalase mimetic activity of nanoceria [3, 4]. Interestingly, CNP at concentrations of 150–300 μM show on one hand a selective antioxidative property in normal (healthy) cells protecting these cells against oxidative impacts such as paraquat or hydrogen peroxide, and on the other hand CNP show a prooxidative cytotoxic activity in tumor cells [5–7]. These unique features point to a promising therapeutic potential of CNP for further in vivo studies in the near future [1]. Toxic and protective effects of nanoceria were found to depend on their preparation method, particle size, cell type and exposure route [8, 9].

Redox homeostasis is often changed in tumor cells and therefore provides a potential target in anticancer therapy. Aside from being toxic in skin tumor cells [10, 11], it has been shown that CNP induce cytotoxicity in human adenocarcinoma SMMC-7721 cells via oxidative stress and the subsequent activation of MAPK signaling pathways [12]. Furthermore, nanoceria induce a dose-dependent increase in the formation of reactive oxygen species (ROS) in A549 lung carcinoma cells leading to a decrease in cellular glutathione (GSH) followed by an induction of apoptosis as determined by elevated expression of Bax, caspase-3, caspase-9 and Apaf1, release of cytochrome c, and a decrease in Bcl-2 expression [13]. In conclusion, most cancer cells exhibit a higher basal ROS level than their non-cancerous counterpart, and it is assumed that this ROS level is increased by CNP up to a level that is specifically toxic for cancer cells [10].

One main source of reactive oxygen species in the cell are mitochondria [14], producing high amounts of superoxide (O_2_^.-^) thereby modulating redox homeostasis [15]. It has been reported that CNP treatment of some cell types resulted in release of cytochrome c. Although it was shown that cerium oxide nanoparticles are co-localized with mitochondria [16] it has not been investigated so far whether CNP mediate mitochondria-triggered ROS formation followed by changes in mitochondrial morphology and/or bioenergetics. Mitochondria, known as the powerhouse of the cell, play an important role in essential processes besides ATP synthesis such as proliferation, differentiation, calcium homeostasis and apoptosis [17, 18]. They form a rapidly changing dynamic network in the cells, that is modulated in an on-going process of fusion and fission [19, 20]. The equilibrium of fusion and fission is often disturbed in mitochondrial and neurodegenerative diseases, in ageing and also in cancer [21–23]. Fusion and fission are part of the mitochondrial quality control [24, 25], and it has been published that morphological and ultrastructural changes, that lead to a disturbed quality control of mitochondria, are often induced by ROS [26, 27]. CNP have been reported to diminish oxidant-induced ROS production in human dermal fibroblasts [6, 7], concomitant with a rescue of mitochondrial membrane potential (MMP) and mitochondrial morphology, i.e. fusion and fission [7]. In tumor cells, however, already elevated ROS level lead to a change in the cellular metabolism and signaling cascade [28, 29], a phenomenon recently described as redox signaling [30–33]. Elevated ROS levels and accordingly altered redox-status makes the cancer cell more vulnerable, thus providing an excellent tool for therapeutic treatment with prooxidant CNP [34]. Hereof we address the question whether the prooxidative property of nanoceria in melanoma cells affects mitochondrial functions. CNP effects were studied in relation to changes of the mitochondrial ROS production and mitochondrial membrane potential, on alterations of the mitochondrial dynamics and morphology, and the ATP content reflecting a bioenergetics parameter. We show that a CNP initiated generation of H_2_O_2_, acted directly at a mitochondrial level, and impaired mitochondrial structure and functions. This indicates that the SOD-mimetic activity of CNP does not only lead to a decrease in cancer cell viability but also induces a mitochondrial dysfunction.

## 2. Materials and methods

All chemicals including cell culture medium (Dulbecco´s modified Eagle´s medium (DMEM)) as well as the catalase-polyethylene glycol (PEG-catalase) were obtained from Sigma or Merck (Darmstadt, Germany) unless otherwise stated. The fetal bovine serum was from Pan Biotech (Aidenbach, Germany). The Protein Assay Kit (Bio-Rad DC, detergent compatible) was from Bio-Rad Laboratories (Feldkirchen, Germany). The enhanced chemiluminescence system (SuperSignal West Pico/Femto Maximum Sensitivity Substrate) was supplied by Pierce (Fisher Scientific, Schwerte, Germany). Penicilin/Streptomycin was obtained from Biochrom (Berlin, Germany) and Glutamax from Gibco (Darmstadt, Germany). MitoTEMPO was purchased from Enzo Life Sciences (Lausen, Schweiz). MitoSOX Red and MitoTRACKER Green FM were obtained from Fisher Scientific (Schwerte, Germany). Beta-actin antibody was purchased from Cell Signaling (Massachusetts, USA). The polyclonal rabbit α-hapten antibody directed against dimedone tagged sulfenic acids was kindly provided from K.S. Carroll [35]. TOM-20 antibody was purchased from Santa Cruz Biotechnology (Dallas, USA). DMSO was obtained from Roth (Karlsruhe, Germany). Horseradish peroxidase (HRP) conjugated goat anti-rabbit IgG from Dianova (Hamburg, Germany) and HRP conjugated rabbit anti-mouse IgG from Dako (Glostrup, Denmark) were used as a secondary antibody.

### Cell culture

The human malignant melanoma cell line A375, originally derived from a 54-year-old woman, was purchased from ATCC, Virginia, USA (ATCC® CRL-1619^™^). Normal human epidermal melanocytes (NHEM), originally derived from the epidermis of juvenile foreskin or adult skin, were purchased from PromoCell (Heidelberg, Germany) (C-12400). A375 were cultured in low glucose DMEM (Sigma, Darmstadt, Germany) supplemented with 10% FBS (FBS Premium, Pan Biotech). For treatment with CNP, cells were cultured in serum-free high glucose DMEM (Sigma). NHEM were cultured in melanocyte growth medium (C-24010) with Supplementmix C-39415 (PromoCell/Bio-Connect, Huissen, Netherlands). For treatment with CNP cells were cultured in melanocyte growth medium (PromoCell/Bio-Connect, Huissen, Netherlands). Both melanoma cells and melanocytes were grown to subconfluence (about 80% confluence) prior to use.

### Cerium oxide nanoparticles

A water-based suspension of Ce IV nanoparticles (CeO_2_, 1.5 mg/ml) was purchased from Sciventions (Toronto, Canada). The nanoparticles are stabilized in sodium polyacrylate (1.27 mg/ml) and had a mean diameter of 1–10 nM [9].

### Cell viability assays

#### MTT assay

The cytotoxic effects of CNP and Mito-TEMPO were measured by the MTT (3-(4,5-dimethylthiazol-2-yl)-2,5-diphenyltetrazolium bromide) assay [36]. When indicated, 5000 U/ml catalase were added to the cells 24 h prior to CNP treatment, in this case, cells were washed before adding the nanoparticles. The activity of mitochondrial dehydrogenase, as indicator of cellular viability, results in formation of a purple formazan dye. Briefly, MTT solution (0.5 mg/ml) was added to the cell cultures treated for various times with the nanoparticles or with MitoTEMPO. The cells were incubated for an additional hour. The medium was removed and the cells were lysed in dimethyl sulfoxide. The formazan formation was measured at 570 nm with a FLUOstar OPTIMA plate reader (BMG Labtech, Ortenberg, Germany). The results were presented as percentage of mock-treated control which was set at 100%.

#### Sulforhodamine B (SRB) assay

The cytotoxic effects of CNP were also measured by the Sulforhodamine B assay, which is based on the pH dependent staining of total proteins using SRB as dye [37]. The assay was performed using 24 well plates. Briefly, after incubation with CNP, the medium was removed and the cells were washed 1x with PBS, fixed with a TCA solution (10% w/v) for 60 min at 4°C, washed 5x with dH_2_O, and dried overnight at RT. After staining with 300 μl SRB solution (0.4% w/v in 1% acetic acid) for 15 min at RT, the cells were washed 5x with 1% acetic acid and dried at RT. For extraction of the dye, 400μl TRIS-Base (10 mmol/l) was added and the plate was gently rotated for 5 min. Finally, the absorbance was measured at 492 nm against the background at 620 nm with a microplate reader. The untreated control was set to 100%.

### SDS-PAGE and Western blotting

SDS-PAGE was performed according to the standard protocols published elsewhere [38] with minor modifications. Briefly, cells were lysed after incubation in 1% SDS with 1:1000 protease inhibitior cocktail (Sigma, Taufenkirchen, Germany). After sonication, the protein concentration was determined by using a modified Lowry method (DC^™^ Protein Assay Kit, Bio-Rad, California, USA). 4x SDS-Page sample buffer (40% glycerol, 20% beta-mercaptoethanol, 12% SDS, 0.4% bromphenol blue) was added, and after heating the samples (20 μg total protein/lane) were applied to 12% (w/v) SDS-polyacrylamide gels. After electroblotting, immunodetection was carried out (1:1000 dilution of primary antibodies, 1:20,000 dilution of secondary antibody). Antigen-antibody complexes were visualized by an enhanced chemiluminescence system. Beta-actin and TOM-20 were used as internal control for equal loading.

### Measurement of intracellular ROS

Generation of ROS was determined using 2´,7´-dichlorodihydrofluorescein diacetate (H_2_DCF-DA), a widespread used dye [39, 40] that diffuses across the lipid membrane into cells and is subsequently oxidized by intracellular ROS forming the highly fluorescent DCF. Subconfluent A375 oder NHEM were exposed to 300 μM CNP in 24-well plates. Untreated subconfluent cells were used as negative controls. Medium was substituted after 24 h by 100 μM H2DCF-DA containing Hanks Balanced Salt Solution (HBSS). DCF fluorescence was detected at an excitation wavelenght of 485 nm and emission wavelength of 520 nm for 90 minutes in 5 min intervals in a FLUOstar OPTIMA plate reader (BMG Labtech).

### MitoSOX and MitoTRACKER fluorescence staining

Generation of superoxide in mitochondria was determined using MitoSOX Red fluorescent probe (Thermo Fisher Scientific), a lipophilic triphenylphosphonium cation containing dye that diffuses across the lipid membrane and selectively accumulates in mitochondria, where a red fluorescence is produced upon oxidation by superoxide [41]. To label mitochondria, the cell-permeant MitoTRACKER Green FM probe was used. MitoTRACKER contains a thiol reactive moiety and accumulates in the mitochondrial matrix covalently binding to mitochondrial proteins [42].

### Determination of oxidized thiol groups (sulfenic acid)

A375 melanoma cells were grown to subconfluence on tissue culture dishes. After removal of serum-containing medium, cells were cultured in serum-free medium and either mock-treated or treated for 4h (immunostaining) or 4 and 24 h (Western blot) with 300 μM cerium oxide nanoparticles. For the last 2 h of incubation 10 mM dimedone was added. As positive control, the cells were co-incubated with 10mM dimedone and 1 mM H_2_O_2_ for 2 h. After incubation with CNP, cells were either fixed for an immunochemical staining or harvested and washed with PBS. The staining was performed by using the α-hapten antibody raised against the oxidation product of sulfenic acid and dimedone [35]. Nuclei were stained with DAPI. For Western blot analysis, after harvesting cells were fractioned into cytosolic and mitochondrial extracts by crude mitochondria isolation. Briefly, cells were washed with PBS and then scrapped in PBS and centrifuged for 5 min at 500xg; after that the pellet was resuspended in cold lysis buffer (210 mM Mannitol, 70 mM Sucrose, 1 mM EDTA, 20 mM HEPES, protease inhibitor) for 10 min on ice. Then cells were homogenized using a syringe with a 20G needle, followed by centrifugation at 600xg, 4°C for 10 min; supernatant needs to be turbid. The cells were centrifuged again at 1000xg, 4°C for 10 min and the supernatant was transferred to a new tube. Differential fractionation was continued according to Wieckowski et al. [43] with a centrifugation at 6500xg, 4°C for 15 min. The pellet (contains the mitochondrial fraction) was resuspended in lysis buffer and the protein concentration was determined. The fractions were analyzed with the α-hapten (1:1000) antibody directed against oxidized thiol groups (e.g. R-SOH).

### Tetramethylrhodamine (TMRM) staining

To determine mitochondrial membrane potential, A375 cells were incubated with or without CNP, washed with PBS and afterwards loaded with 100 nM of the cell-permeant and membrane potential sensitive dye tetramethylrhodamine methyl ester (TMRM) and 100 nM of the membrane potential insensitive dye MitoTRACKER^™^ Green for 30 min. TMRM is a fluorescent potentiometric and cationic-lipophilic dye that accumulates in healthy (functioning) mitochondria, resulting in a bright fluorescence signal. Upon loss of the mitochondrial membrane potential by apoptotic processes or other stressors, the fluorescence signal is weakened or completely disappears [44, 45]. A concentration of 10 μM carbonyl cyanide m-chlorophenyl hydrazone (CCCP), an oxidative phosphorylation uncoupler [46], served as positive control. After incubation, the cells were washed with PBS once and fresh medium was added. Image stacks were acquired and at least 20 image stacks per sample were analyzed. The membrane potential is given as the fluorescence of TMRM divided by the fluorescence of MitoTRACKER ^™^ Green for normalization.

### Mitochondrial fragmentation

A375 cells were cultured to subconfluence on tissue culture dishes and treated or not with 300 μM CNP or 10 μM CCCP for 24 h. Thereafter, cells were fixed and immunostained against cytochrome *c* and DAPI. Merged confocal fluorescence images were used for mitochondrial morphology quantification in cells as described earlier [47]: *tubular*, at least one mitochondrial tubule of 5 μm or more; *intermediate*, at least one tubule between 0.5 and 5 μm; *fragmented*, no tubules of more than 0.5 μm in length.

### Measurement of total ATP by UPLC

Cells were seeded on 24-well plates and cultured to approximately 70% confluency. After treatment, plates were put on ice and washed 3x with ice cold PBS. Lysis was done with an ice cold, acidic solvent containing acetonitrile, ddH_2_O and trifluoracetic acid (80/19/1) (v/v) at a volume of 150 μl/well. Cells were harvested into an 0.5 ml tube and centrifuged at 21,000xg for 10 min at 4°C. After that, the liquid phase was transferred to a 200 μl tube and evaporated. The precipitate was dissolved in 50 μl running buffer A (200 mM KH_2_PO_4_/200 mM KCl, at pH 6). Equal amounts were injected for analysis. UPLC analysis was performed using Water´s Acquity Ultra Performance Liquid Chromatographic system H class (Waters Corp., Milford; MA USA). Analytes were separated by running a linear gradient of buffer A (200 mM KH_2_PO_4_/200 mM KCl, at pH 6) and buffer B (200 mM KH_2_PO_4_/200 mM KCl/7.5% acetonitrile at pH 6) with a flow profile of 0.340 ml/min as followed: initial 100% A; 0.03 min 96% A, 4% B; 4.53. min 91% A, 9% B; 22.63 min 5% A, 95% B; 26.10 min 5% A, 95% B; 26.50 min 100%A. The UPLC separation was done using a Cortecs C18 UPLC column (3.0x150 mm, 1.6 μM) (Waters Corp., Milford; MA USA) as stationary phase. UV data were collected at 254 nm. Sample volumes of 10 μL were injected by a cooling autosampler. After the analysis, column was washed and thereby re-equilibrated using buffer A (100%) at a flow rate of 0.34 ml/min. For cleaning, the column was washed with buffer C (ddH_2_O/0.2% Methanol) after every experimental series. To quantify sample concentrations, area under curve was integrated using Empower 3 software (Waters) and calculation was done via a known standard concentration corresponding to the analytes.

### Transmission electron microscopy for cristae quantification

A375 melanoma cells were mock-treated or incubated with CNP for 4 or 24h and analyzed via electron microscopy. For this, the cells were grown on culture dishes up to 80% confluency. TEM samples then were fixed for a minimum of 4 h in 2.5% v/v glutaraldehyde (GA) and 4% w/v paraformaldehyde (PFA) in 0.1 M cacodylate buffer (pH 7.4) at 4°C. Then, samples were incubated in 1% osmium tetroxide in 0.1 M cacodylate buffer for 2 h. Dehydration was achieved using acetone (50%, 70%, 90% and 100%) and block contrast was applied (1% phosphotungstic acid/0.5% uranylacetate in 70% acetone). A SPURR embedding kit (Serva, Heidelberg, Germany) was used to embed samples, which were polymerized overnight at 70°C, before cutting into 80 nm sections using an Ultracut EM UC7 (Leica, Wetzlar, Germany). Images were captured using an H600 TEM (Hitachi, Tokyo, Japan) at 75 kV. Number of cristae and diameter of mitochondria were determined for 30 mitochondria; then the mean number of cristae was normalized to the mean of mitochondrial diameter.

### PEG-catalase incubation

Catalase-polyethylene glycol was obtained from Sigma (Darmstadt, Germany). A375 cells were grown on culture dishes up to 80% confluency and preincubated or not with 5000 U/ml of PEG-catalase for 4 h. Then the cells were washed and mock-treated or incubated with CNP for 96 h. Viability was assessed as described above.

## 3. Results

### 3.1. CNP selectively lower the viability of tumor cells compared to normal cells

**Fig 1:**
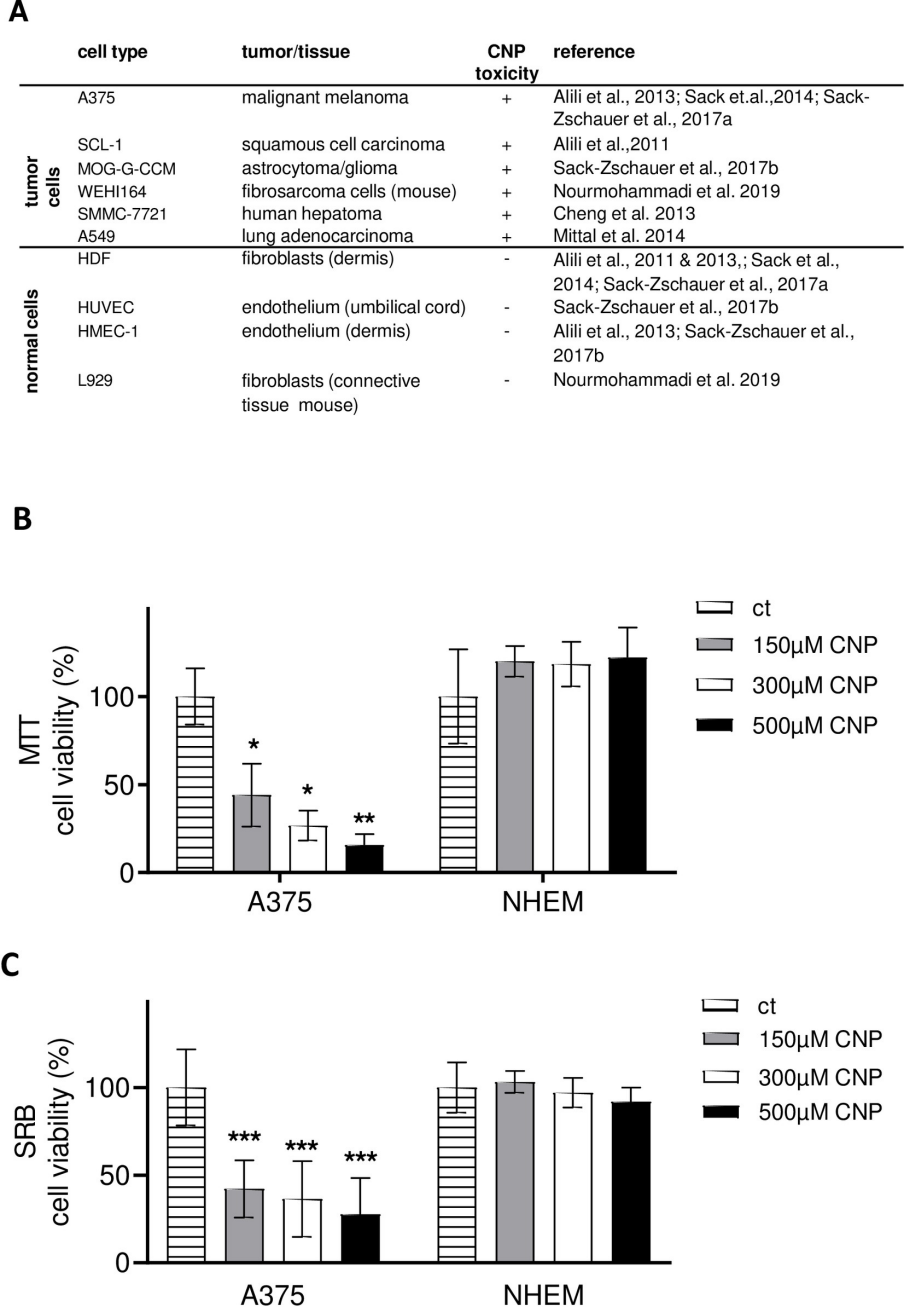
CNP are toxic to tumor cells but not to normal cells. A, the table summarizes recent studies from our lab and others on the toxicity of nanoceria in different cell types. B and C, CNP (96 h) dose-dependently decreased cell viability in A375 malignant melanoma cells, but not in normal human epidermal melanocytes (NHEM), measured with the MTT (B) or SRB (C) assay. The percentage of cell viability of the mock-treated control (ct), which was set at 100%, is presented. ANOVA with Dunnett´s post-hoc test was used for the determination of statistical significance. Data are means ± SEM, n = 3–6. *, p < 0.05; **, p<0.01 and ***, p<0.005 compared to control.

In earlier in vitro studies, it has been shown by others and our group that CNP treatment lowers the viability of various tumor cell lines while not affecting the viability of non-cancerous cells (Fig 1A). Here we studied the human malignant melanoma cell line A375 and normal human epidermal melanocytes (NHEM). Increasing concentrations of CNP dose-dependently and significantly lowered the viability of A375 cells compared to the mock-treated controls, whereas CNP had no statistically significant toxic effect on melanocytes (Fig 1B and 1C) at 96 h post treatment. The MTT assay is based on the measurement of the metabolic activity of the cells. It quantifies viable cells through their ability to reduce 3-(4,5-dimethylthiazol-2yl)-2,5-diphenyltetrazolium bromide, a soluble yellow tetrazolium salt, to a blue-purple formazan precipitate. This requires the activity of the mitochondrial succinate dehydrogenase [48, 49]. As the formazan used in the MTT assay per se is cytotoxic over time (resulting in false negative or positive results), a second viability assay, the SRB assay, was used to verify the MTT data. SRB is an anionic dye, which binds to basic amino acid residues in fixed cells to provide a sensitive index of cellular proteins [50]. Both assays showed in tendency the same results (Fig 1B, MTT; Fig 1C, SRB). For further experiments, a concentration of 300 μM CNP was used as a standard concentration which was already used earlier [51].

### 3.2. A higher basal (mitochondrial) ROS level in melanoma cells compared to melanocytes makes them more sensitive to exogenous H_2_O_2_

**Fig 2:**
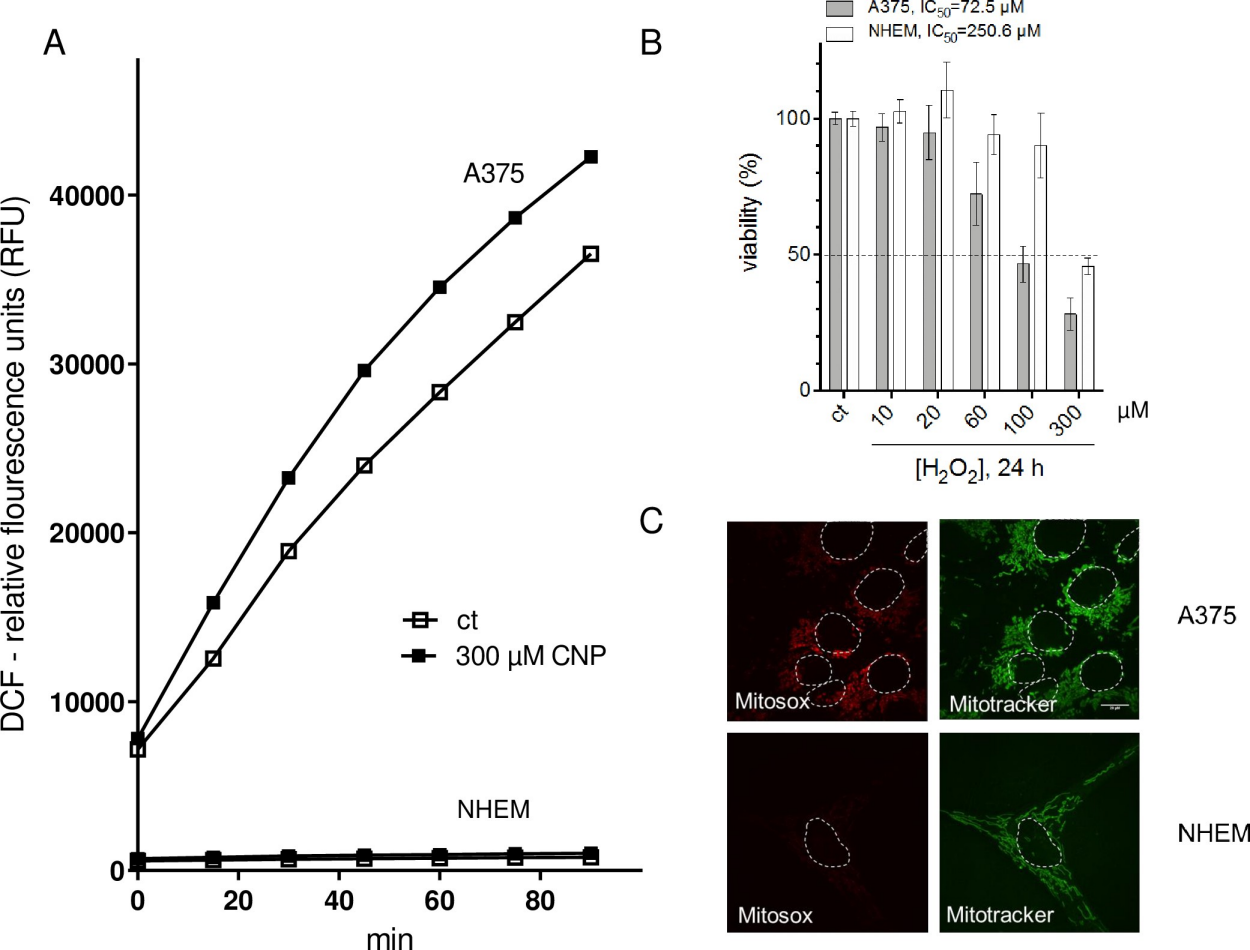
A higher basal (mitochondrial) ROS level in melanoma cells compared to melanocytes makes them more sensitive to exogenous H_2_O_2_. A, ROS formation in presence and absence of CNP in melanoma cells and melanocytes was determined by relative fluorescence of DCF. B, Hydrogen peroxide (24 h) dose-dependently reduced the viability of both A375 and NHEM, but with a different IC_50_ value. C, A375 per se exhibited an increased level of mitochondrial superoxide (as indicated by the fluorescent dye MitoSOX (10 μM,10 min) compared to melanocytes. The fluorescent dye Mitotracker (100 nM) was used to visualize mitochondria, and to better distinguish between the cells, nuclei were encircled with a white dotted line.

It was published that tumor cells exhibit higher ROS levels than normal (healthy) cells [10, 28, 29, 51] and that CNP may exhibit prooxidative as well as antioxidative properties [5, 6, 52]. This nanoparticles behave as superoxide dismutase (SOD) mimetics [3, 53] and are therefore able to decrease the cellular amount of superoxide while increasing the amount of hydrogen peroxide [5, 10]. To clarify the prooxidative (and/or antioxidative) effect of CNP in A375 melanoma cells and human melanocytes, being the physiological counterpart, both cell types were incubated with 300 μM CNP for up to 90 minutes. Fig 2A shows the relative fluorescence of DCF, a fluorogenic dye that is commonly employed to represent ROS formation. A375 cells exhibit a significant higher ROS level than the melanocytes indicated at time point 0 minutes. This ROS amount is even increased by treatment with nanoceria. CNP do not affect the ROS amount in NHEM over the studied time period.

We speculate that CNP induce higher hydrogen peroxide amounts via their SOD mimetic activity in melanoma cells compared to melanocytes due to an already higher basal ROS level in the tumor cells. To address this, treatment both cell types with exogenously added H_2_O_2_ should mimic the nanoceria effects in a dose-dependent manner, with melanoma cells dying from lower doses of the oxidant than melanocytes. A375 melanoma cells and NHEM were treated with increasing concentrations of exogenous H_2_O_2_ for 24 h (Fig 2B). Indeed, the melanoma cells showed a lowered viability compared to normal cells with increasing H_2_O_2_ concentrations, confirming that these cells are more sensitive to an additional oxidative provocation. These data are reflected by the IC_50_ values calculated by nonlinear curve fit analysis and evaluation of goodness-of-fit (all runs tests >0.5, all R^2^ >0.9) as described [54]. The IC_50_ value for H_2_O_2_ treated A375 melanoma cells was calculated to be 72.5 μM and 250.6 μM for NHEM. Together with the results depicted in Fig 2A, it indicates that the higher endogenous basal level of ROS in the tumor cells makes them more susceptible to exogenous oxidative stress.

The SOD mimetic activity of CNP needs superoxide (O_2_^.-^) as "substrate" for H_2_O_2_ production. A major source of superoxide in the cell are the mitochondria [14]; both A375 tumor cells and NHEM were tested for the level of mitochondrial superoxide with MitoSOX, a mitochondrial superoxide indicator [41]. MitoSOX enters the cells and specifically targets to mitochondria. It is oxidized by superoxide to a strong red fluorescent dye, but not by other reactive oxygen or nitrogen species. As expected, untreated A375 melanoma cells in contrast to melanocytes show a significant stronger fluorescence signal with MitoSOX, with MitoTRACKER validating mitochondrial localization (green fluorescence) (Fig 2C). In summary, the high base level of superoxide in the tumor cells may finally lead to the significant stronger DCF signal in these cells, which can be further elevated by the treatment with SOD-mimetic nanoceria (Fig 2A).

### 3.3. Mitochondrial ROS, generated by a mitochondrially targeted SOD mimetic, lower tumor cell viability and increase thiol oxidation

**Fig 3:**
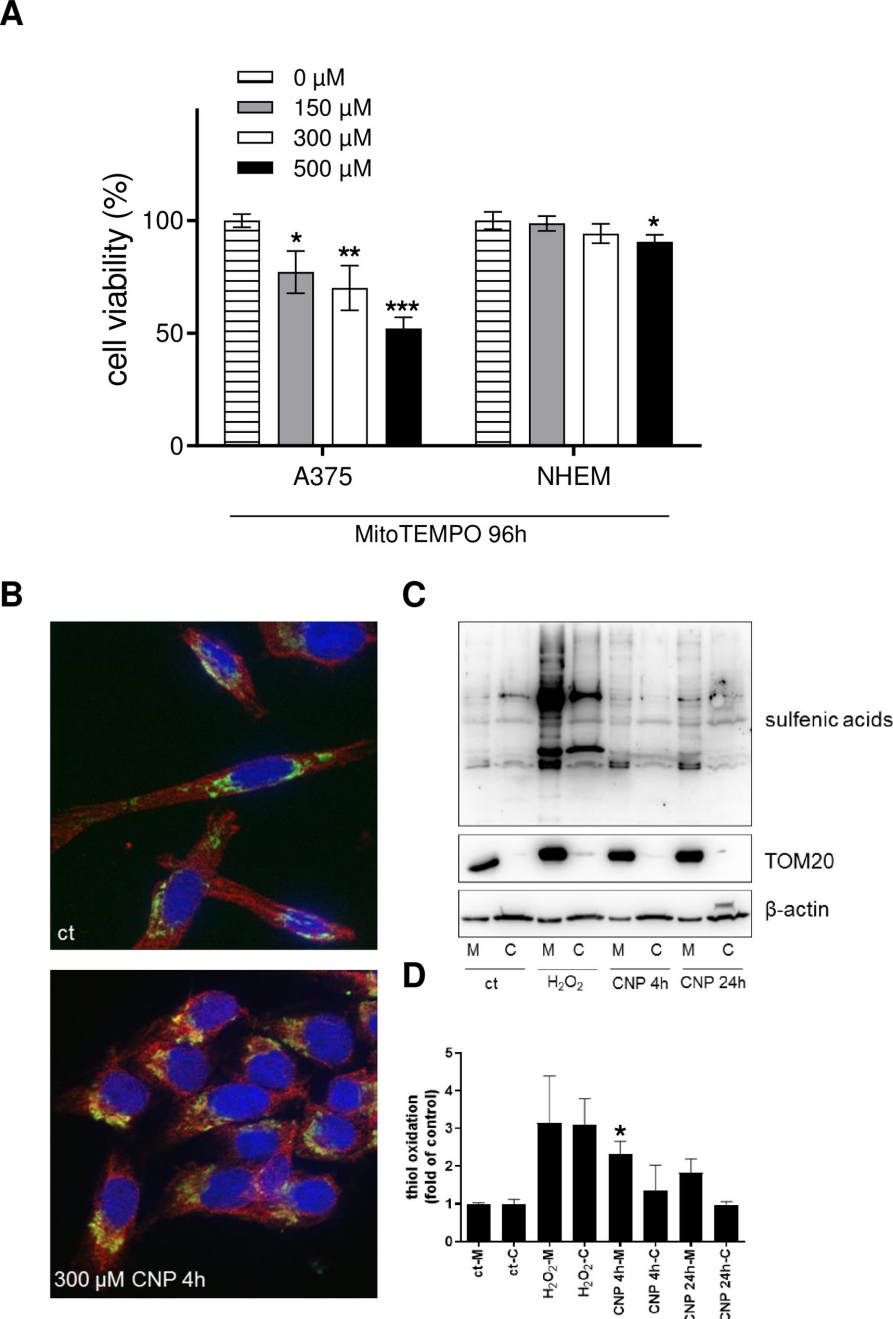
Mitochondrial ROS lower tumor cell viability and increase thiol oxidation. A, MitoTEMPO, a mitochondrial superoxide scavenger that dismutates superoxide to hydrogen peroxide, decreased cell viability in a dose dependant manner after 96 h in A375, but not in NHEM. ANOVA with Dunnett´s post-hoc test was used for the determination of statistical significance. Data are means ± SEM, n = 3–6. *, p<0.05; **, p<0.01; ***, p<0.0001 compared to control. B, Nanoceria (300 μM) significantly increased mitochondrial thiol oxidation as indicated by immunofluorescence (cytochrome c: green; oxidized thiols: red) after 6 h of incubation (CNP 4 h, followed by addition of dimedone and additional 2 h of incubation). C, A375 cells were separated into cytosolic and mitochondrial fraction after mock-treatment, incubation with H_2_O_2_ or incubation with CNP. Western blot analysis revealed an increase in mitochondrial thiol oxidation compared to the cytosolic fraction. A representative Western blot of 4 individual experiments is shown (n = 4). D, Densitometric analysis of Western blot results of panel C. Thiol oxidation was determined relative to ß-actin and TOM20 signal for cytosol and mitochondria, respectively. The thiol oxidation was depicted as fold of control, which was set at 1. ANOVA with Tukey´s post-hoc test was used for the determination of statistical significance between the mitochondrial fractions of mock-treated and CNP-treated cells.

As the viability of cells is dependent on proper mitochondrial function [55, 56], and MitoSOX staining hints to a higher superoxide (O2^.-^) level in tumor cell mitochondria vs normal cell mitochondria (Fig 2C), the results thus far indicate a pivotal role of mitochondria in the selective toxicity of CNP. To test the hypothesis that an increased ROS amount within the mitochondria is (at least in part) responsible for the CNP mediated toxicity in tumor cells, A375 and NHEM were incubated with MitoTEMPO, a mitochondrially targeted [57] antioxidant. This compound is also able to pass biological membranes and because of its positive charge it accumulates within mitochondria driven by the membrane potential. Once localized in the mitochondria it dismutates mitochondrial superoxide (via its SOD mimetic activity) and thereby elevates the level of (mitochondrial) hydrogen peroxide. According to our results concerning the toxicity of the SOD mimetic nanoceria (Fig 1B) and of rising H_2_O_2_ concentrations (Fig 2B), we expected that MitoTEMPO also causes toxicity in melanoma cells but not in melanocytes. Indeed, a significant dose-dependent decrease of A375 viability to approximately 50% was measured after 96 h compared to mock-treated controls (ct) (Fig 3A). In contrast, only a slight effect of MitoTEMPO on non-cancerous NHEM was observed, whereby viability was lowered only by the highest concentration of 500 μM MitoTEMPO to approximately 85–90% (Fig 3A). In conclusion, these results indicate that a significant superoxide source, required for any SOD mimetic activity of CNP, could be mitochondria.

Concomitant with a higher ROS level in CNP-treated tumor cells, CNP also induced carbonylation and thiol oxidation of cytosolic proteins [5]. As the experiments with MitoTEMPO and MitoSOX indicated a significant higher ROS level in mitochondria of A375 melanoma cells compared to the mitochondria of melanocytes, we checked for thiol oxidation of mitochondrial proteins in melanoma cells after CNP treatment. There is accumulating evidence that cysteine sulfenation (Cys-SOH) or higher oxidation products play an important role in cellular response to oxidative stress [58, 59], and several proteins such as GAPDH, peroxiredoxins and certain phosphatases have been shown to undergo thiol oxidation in cells treated with hydrogen peroxide [60]. Fig 3B shows a representative immunostaining of cytochrome *c* (green) as marker for mitochondria and oxidized thiols (red) in A375 melanoma cells. Nuclei were stained with DAPI (blue). Merging of both red and green fluorescence (yellow) indicate colocalization of oxidized thiols and cytochrome *c* and thus oxidation of thiols groups located in mitochondria. To validate the staining results, the same experiment, i.e. CNP treatment of A375 melanoma cells, was repeated for Western blot analysis. Here, we checked for 2 time points of CNP incubation, to cover both an early and a later timepoint in the initial phase of CNP toxicity. After treatment, cell compartments were separated into cytosolic and mitochondrial fractions. The Western blot shown in Fig 3C is representative for 4 independent experiments and confirms the results obtained from cellular staining (Fig 3B). Both a 4 h and 24 h incubation with 300 μM nanoceria induced oxidation of thiol groups in the mitochondria as well as in the cytosol (Fig 3C). Compared to hydrogen peroxide that induced thiol oxidation in both fractions and served as a positive control [61, 62], CNP showed a tendency of more oxidation in the mitochondrial fraction compared to the cytosolic fraction (Fig 3D).

### 3.4. CNP significantly decrease mitochondrial membrane potential and induce mitochondrial fragmentation only in melanoma cells

**Fig 4:**
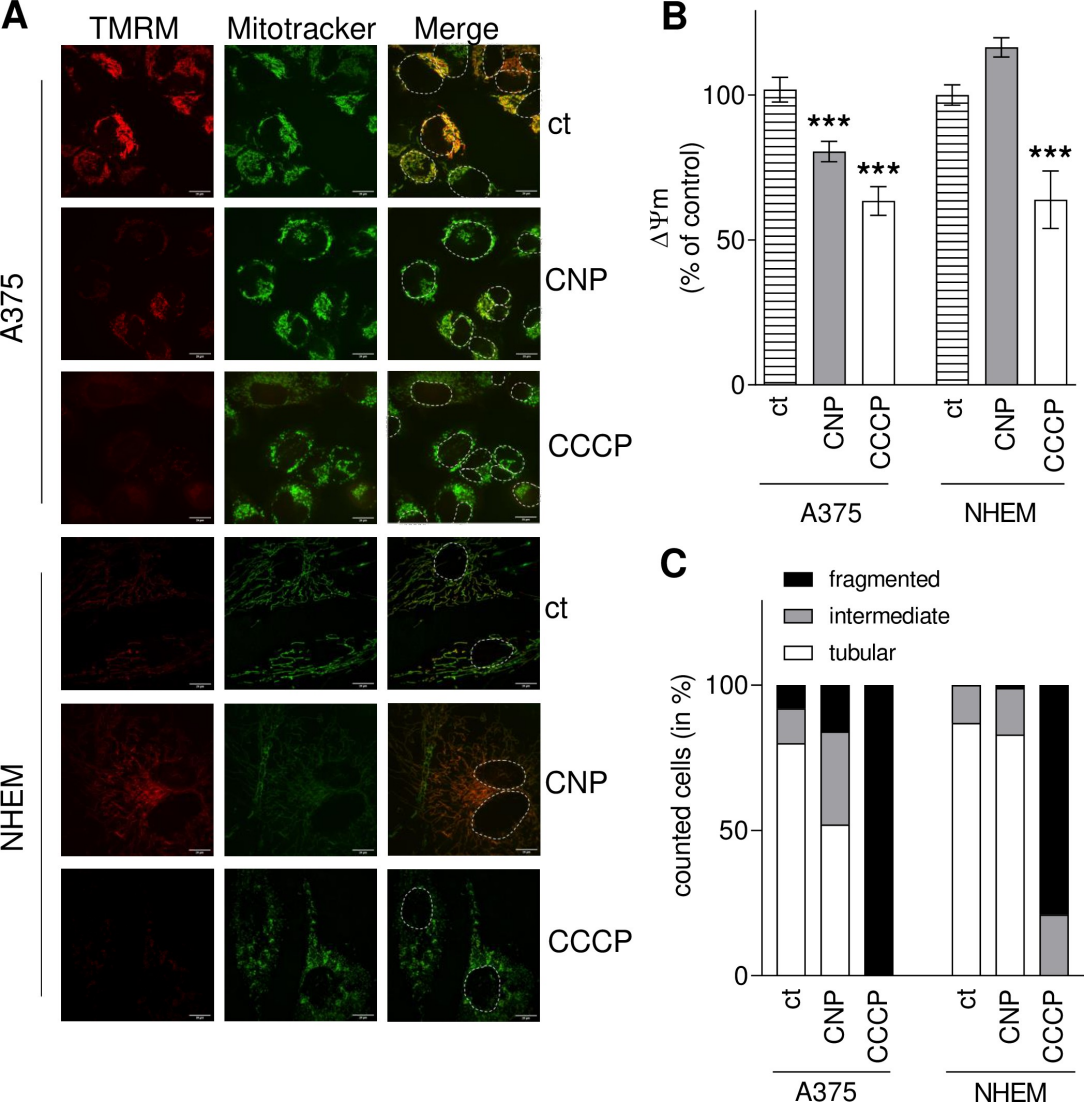
CNP decrease mitochondrial membrane potential and induce mitochondrial fragmentation in melanoma cells. A, 300 μM CNP (4 h) significantly decreased mitochondrial membrane potential in A375 cells, but not in melanocytes, indicated by changes of TMRM dependent fluorescence in mitochondria. A 2 h treatment with CCCP served as control. The white lines indicate 20μm, and to better distinguish between the cells, nuclei were encircled with a white dotted line. B, Image analysis of TMRM fluorescence. To normalize the results, the fluorescence intensity of TMRM was divided by the fluorescence intensity of MitoTRACKER. ANOVA with Dunnett´s post-hoc test was used for the determination of statistical significance. Data are means ± SEM, n = 3. ***, p < 0.0001 compared to mock-treated control (ct). C, Quantification of melanoma and melanocyte mitochondrial morphology after treatment with CNP or CCCP for 24 h according to Duvezin-Caubet et al. [47]: tubular (>5 μm), intermediate (0.5–5 μm), fragmented (< 0.5 μm). Experiments were performed in triplicate (n = 3) with analysis of 50 cells each treatment and each experiment.

Furthermore, it was reported that an increased mitochondrial ROS level lowered the mitochondrial membrane potential (MMP) [63, 64]. MMP is an essential component in the process of energy storage during oxidative phosphorylation, and a drop in MMP induces loss of cell viability [65]. To assess the impact of CNP on mitochondrial membrane potential, A375 melanoma cells and normal melanocytes were treated with nanoceria or mock-treated (control, ct) and afterwards stained with the cationic-lipophilic dye TMRM, showing a bright fluorescence if an intact MMP exists [45]. MitoTRACKER, a membrane potential insensitive dye, was used again to stain mitochondria. The protonophore carbonyl cyanide m-chlorophenyl hydrazone (CCCP) causes full loss of MMP and therefore was used as positive control. Fig 4A shows representative live cell images of the performed experiments, indicating that both CNP and CCCP induced a loss in the mitochondrial membrane potential in melanoma cells, whereas in NHEM the cerium oxide nanoparticles did not interfere. This was confirmed by a quantitative image analysis shown in Fig 4B. CNP significantly lowered TMRM dependent fluorescence in melanoma cells. It dropped down to 75–80% compared to mock-treated tumor cells (ct) indicating that MMP was lowered by CNP. In contrast, the MMP of the melanocytes was not significantly affected after CNP treatment. As expected, CCCP significantly lowered MMP in both cell types.

Changes in redox homeostasis and a decrease of the MMP often lead to disturbance of mitochondrial dynamics [66, 67], and especially hydrogen peroxide has been reported to induce mitochondrial fission in various cells [68]. As we propose that CNP act via hydrogen peroxide formation due to their SOD mimetic function, we investigated the impact of CNP on mitochondrial fragmentation, i.e. fusion and fission dynamics of the organelle. A375 cells and NHEM were incubated with 300 μM CNP or 10 μM CCCP for 24 hours. After fixation, the cells were immunostained for cytochrome *c* (mitochondria) and DAPI (nuclei). The quantification of mitochondrial morphology (Fig 4C) following the classification described by Duvezin-Caubet et al. [47] revealed that in melanoma cells CNP increased the number of intermediate (grey bar) and fragmented (black bar) mitochondria about 2-fold compared to control (Fig 4C). In contrast to melanoma cells, CNP did not alter the dynamics of mitochondria in melanocytes (Fig 4C). CCCP is known to induce mitochondrial fission and served as positive control for both cell types.

### 3.5. CNP treatment decreases the amount of total ATP in melanoma cells

**Fig 5:**
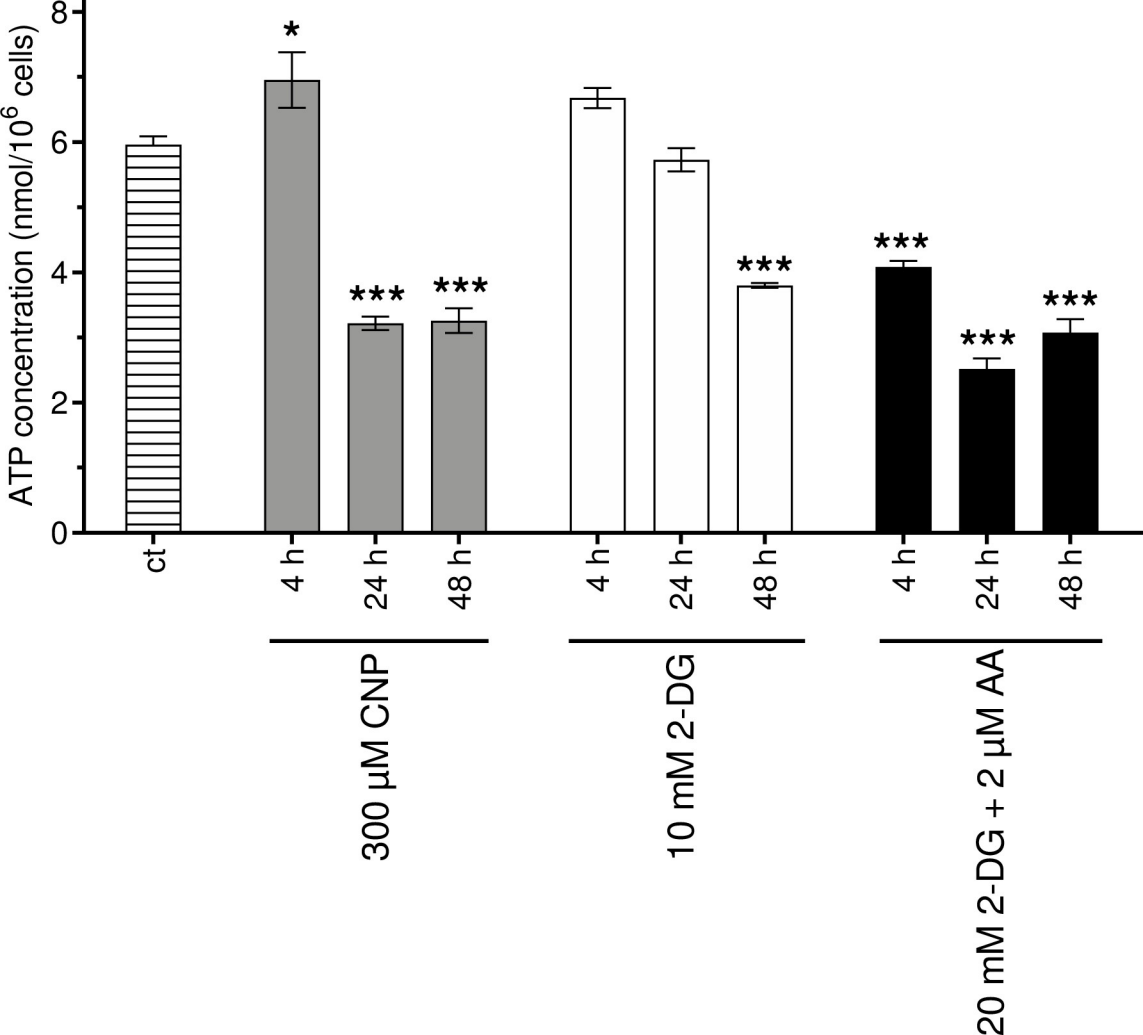
Total cellular ATP is decreased in melanoma cells after CNP treatment. Melanoma cells were incubated with 300 μM CNP, 10 mM 2-deoxy-D-glucose (2-DG) and 20 mM 2-DG in combination with 2 μM antimycin-A (AA) for 4, 24, and 48 h or untreated (ct) for 4 h. The amount of total ATP was assessed via UPLC and calculated by division of the total amount of ATP per well by cell number. ANOVA with Tukey´s post-hoc test was used for the determination of statistical significance. Data are means ± SEM, n = 3. *, p < 0.05; ***, p < 0.0001 compared to mock-treated control (ct).

Recently, cerium oxide nanoparticles have been shown to protect primary cultured skin fibroblasts from hydrogen peroxide induced damage [7]. This is in line with earlier published data [6], showing that nanoceria protect human dermal fibroblast from another oxidative insult, namely paraquat. Pezzini et. al. [7] evaluated mitochondrial function in mock-treated cells and after treatment with hydrogen peroxide. In addition to the observed nanoceria mediated protection of the fibroblasts against H_2_O_2_ initiated loss of mitochondrial membrane potential and change of the mitochondrial dynamics, nanoceria also protected against the a H_2_O_2_ mediated decrease of the total ATP amount. As we propose that nanoceria, albeit being antioxidative in normal (healthy) cells, exhibit prooxidative properties inducing a higher amount of hydrogen peroxide in A375 melanoma cells, we also checked for total ATP amount in A375 after CNP treatment. Indeed, nanoceria at a concentration of 300 μM lowered the amount of total ATP in the cell after 24 h. After a 48 h treatment with CNP, the total ATP content did not further decline. The glucose analogue 2-deoxy-D-glucose (2-DG) is a compound that interferes as 2-DG-6-phosphate with the carbohydrate metabolism e.g. by inhibiting glycolytic enzymes such as glucose-6-phosphate isomerase [69]. Antimycin A (AA) is used as an inhibitor of complex III of the electron transport chain (ETC), thereby inhibiting oxidative phosphorylation (OXPHOS) [70]. Both 2-DG and AA served as positive controls. A time dependent decrease of the ATP amount after treatment with 10 mM DG could be enhanced by a combination with 2 μM AA (Fig 5).

### 3.6. The number of cristae in melanoma cell mitochondria is decreased upon CNP treatment

**Fig 6:**
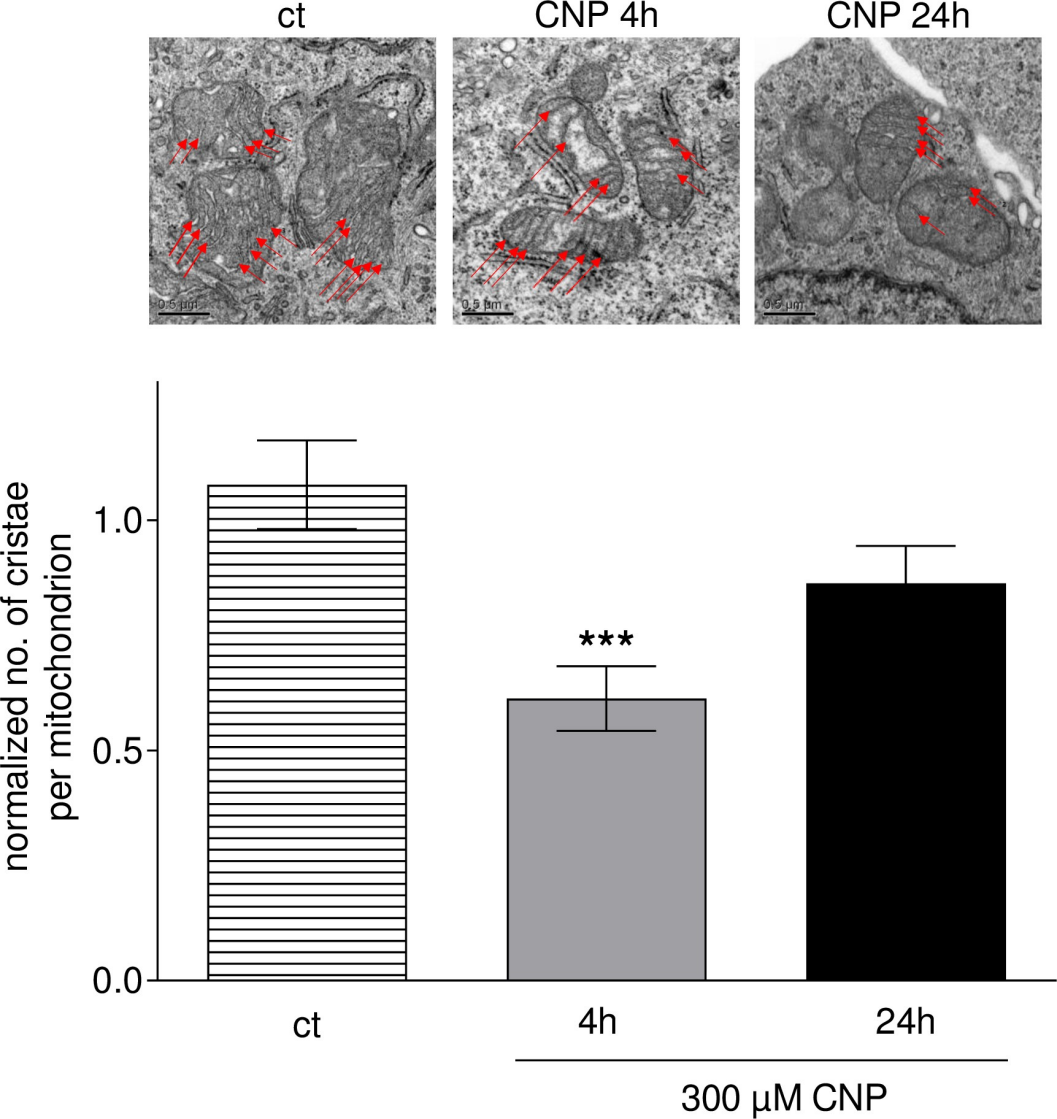
CNP decrease the number of cristae in melanoma cells. Melanoma cell were treated with 300 μM CNP for 4 and 24 h or mock-treated (ct). Transmission electron microscopy for cristae quantification was performed as described in the Methods section. CNP significantly decreased the number of cristae in the mitochondrial inner membrane in A375 cells. ANOVA with Dunnet´s post-hoc test was used for the determination of statistical significance among treatment groups. Data are means ± SEM, n = 3. ***, p < 0.0001.

ATP production mainly takes place via the ETC located in the inner mitochondrial membrane. Mitochondrial cristae are the folds within this membrane, and these folds allow an increased surface in which the reactions of the ETC can take place. The surface area of cristae positively correlates with the amount of ATP produced by oxidative phosphorylation [71]. As incubation with CNP lowered the total ATP amount in the melanoma cells (Fig 5), we counted the number of cristae in these cells after CNP treatment. Upon a 4 h incubation with 300 μM CNP, the number of cristae per mitochondrion significantly decreased to about half of the mock-treated control (ct) (Fig 6). This reduction was less evident upon a 24 h treatment compared to the 4 h values.

### 3.7. PEG-catalase rescues melanoma cells from CNP induced cell death

**Fig 7:**
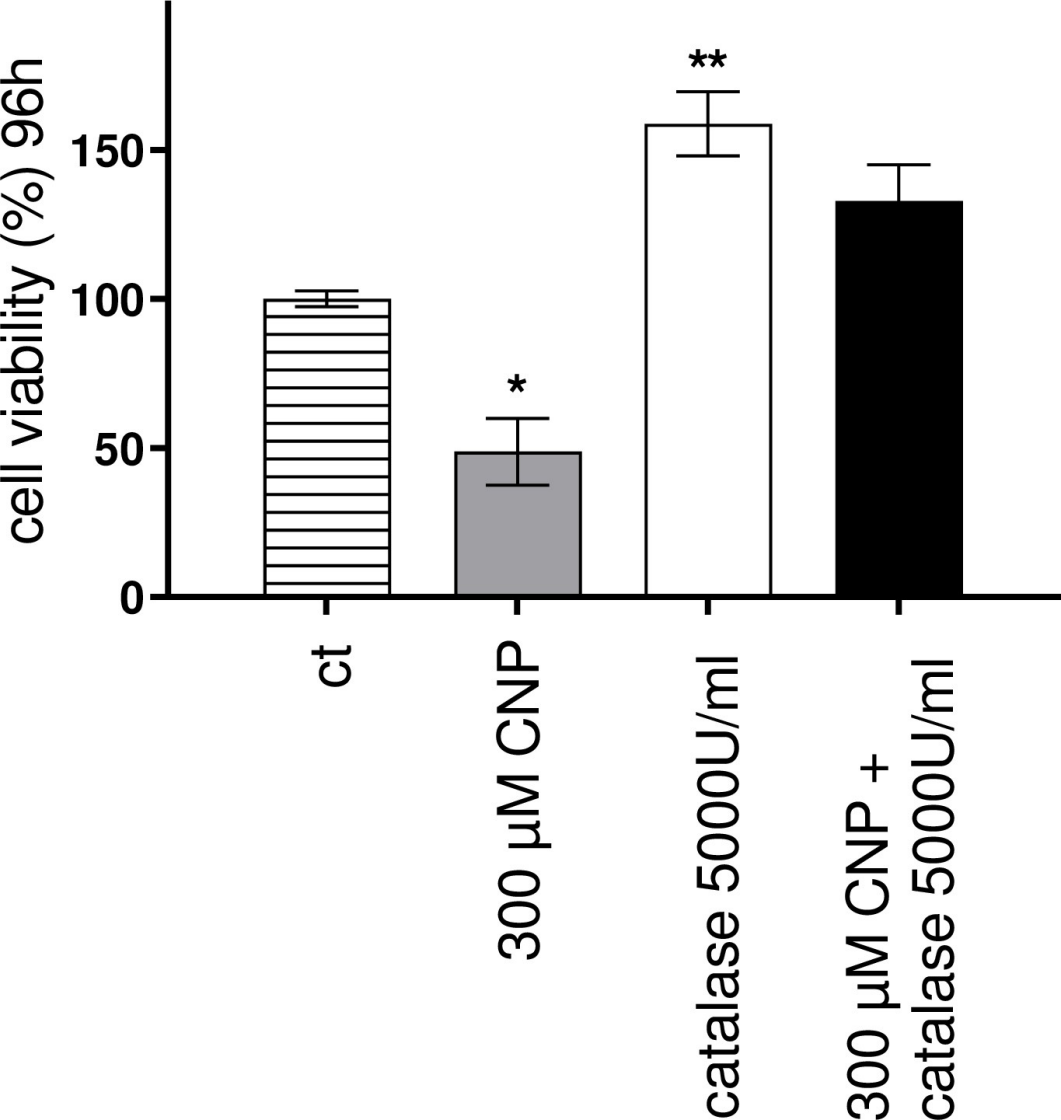
PEG-catalase rescues melanoma cells from CNP induced cell death. Preincubation of A375 tumor cells with PEG-catalase (5000 U/ml) for 4 h fully restored viability of melanoma cells upon a 96 h CNP incubation. ANOVA with Dunnet ´s post-hoc test was used for the determination of statistical significance among treatment groups. Data are means ± SEM, n = 3. ***, p < 0.0001.

Next we wanted to test the hypothesis that CNP induced hydrogen peroxide formation was responsible for the negative outcome of CNP treatment of melanocytes. If so, preincubation with catalase should rescue melanocytes from nanoceria induced cell death. Prior to incubation with 300 μM nanoceria for 96 h, the A375 melanoma cells were incubated for 4 h with catalase (5000 U/ml) coupled to methoxy-polyethylene glycol (PEG). Catalase catalyzes the decomposition of H_2_O_2_ into water and oxygen, and the PEG moiety renders the enzyme more stable while maintaining good reactivity [72]. While CNP lowered the viability of the tumor cells to approximately 50%, PEG-catalase preincubation alone increased cell viability compared to control and fully rescued the melanoma cells from CNP-induced toxicity (Fig 7). In conclusion, the selective cytotoxic activity of nanoceria on A375 melanoma cells was clearly shown to be mediated by hydrogen peroxide and to affect several mitochondrial parameters contrary to what was observed in normal (healthy) cells by our group and others [7].

## 4. Discussion

Redox-based therapies, i.e. therapeutic agents that target redox homeostasis in several diseases, are on the rise, as there are cancer cells that are resistant to for example radiation or classical chemotherapeutical approaches [1, 73]. Cerium oxide nanoparticles (nanoceria, CNP) target the cell´s redox homeostasis by two different ways: they possess antioxidant as well as prooxidant activities, depending on circumstances such as particle size, exposure time and/or cell type [8, 9]. In contrast to other chemotherapeutics, it should be emphasized that nanoceria have never been reported to be toxic to normal (healthy) cells, rather they even show a protective effect. In that context, nanoceria rescue human dermal fibroblasts from different oxidative challenges [6, 7]. In this study, the malignant melanoma A375 cells were selectively killed by CNP, in contrast to normal human epidermal melanocytes (Fig 1). The melanoma cells exhibit a higher basal content of ROS both in the cytosol and the mitochondria (Fig 2A and 2C), a phenomenon attributed to various cancer cells [73]. CNP further elevate the ROS level in the tumor cells, which seems to be due to their previously described SOD-mimetic activity [3]. This activity might be able to shift the balance from superoxide to hydrogen peroxide within the cell, with superoxide serving as a prerequisite to generate H_2_O_2_. Although both superoxide and H_2_O_2_ are damaging the cells after surpassing a certain threshold, H_2_O_2_ is more stable and, therefore, considered to be more toxic [74]. If the nanoceria initiated increase of endogenous H_2_O_2_ is responsible for melanoma cell death, the tumor cells should be more sensitive to exogenously supplied H_2_O_2_ than the normal (healthy) cells. Indeed, our data show that exogenous hydrogen peroxide is more toxic to melanoma cells than to melanocytes, which is reflected by the calculated IC_50_ values (Fig 2B). The selective toxicity of the SOD-mimetic MitoTEMPO (Fig 3A), a mitochondria-targeted substance that increases mitochondrial hydrogen peroxide, further supports our hypothesis, that i) the melanoma cells are more susceptible to increased H_2_O_2_ levels in context of cell death, and ii) a major source of the higher ROS level in tumor cells appears to be mitochondria. As described above, basal ROS level is higher in melanoma cells compared to melanocytes (Fig 2). In that context, an increased ROS level and in consequence an altered cellular redox status result in a specific vulnerability of cancer cells, which can be exploited in therapeutic approaches [34, 75]. A prooxidative approach aims to further increase the ROS level of cancer cells to an extent that exceeds survival strategies resulting in increased cytotoxicity [73]. Disturbance of the cellular antioxidant system or treatment with ROS generating agents, subsequently results in a permanent imbalance of the pro- and antioxidative system, which cannot be compensated by the cancer cells, finally can lead to mitochondrial dysfunction, inactivation of redox sensitive molecules, and apoptosis or necrosis. In contrast, normal (healthy) cells are able to counteract increased ROS levels through adaptation of the endogenous antioxidant systems [76, 77]. Whether the disturbance of the antioxidant system in tumor cells is due to, for example, mitochondrial GSH depletion or loss of glutathione peroxidase (GPx) activity still needs to be clarified. Our group did neither find a significant change in cytosolic GSH content nor in GPx expression after nanoceria treatment of fibroblasts [6], but it has been reported that nanoceria induce cytosolic GSH depletion in A549 cancer cells [13].

The major source of superoxide is the electron transport chain in the inner membrane of mitochondria [14], indicating a possible role of mitochondria in the selective toxicity of nanoceria towards various cancer cells. Following these ideas and keeping in mind that cysteine sulfenation (Cys-SOH) or higher oxidation products play an important role in cellular response to oxidative stress [78], we verified thiol oxidation due to nanoceria both in the cytosol and in the mitochondria of melanoma cells (Fig 3B and 3C). As several proteins such as GAPDH, peroxiredoxins and certain phosphatases have been shown to be regulated by thiol oxidation [79], this could be a possible mechanism by which nanoceria alter the cell´s metabolism.

Pezzini et al. [7] reported, that nanoceria increase the total amount of ATP in human dermal fibroblasts and, furthermore, protect the fibroblasts against a H_2_O_2_ mediated mitochondrial damage by decreasing mitochondrial fission and preventing the cells from loss of the mitochondrial membrane potential. A similar protective effect of nanoceria on human dermal fibroblasts have been observed by our group [6]. As we assumed a very different way of interaction of the nanoceria with mitochondria in the melanoma cells, we started to investigate mitochondrial parameters. The used nanoceria actually act the opposite way in melanoma cells: upon treatment a loss of the mitochondrial membrane potential (Fig 4A and 4B), an increase in mitochondrial fission (Fig 4C), and a loss of total ATP (Fig 5) was observed.

Mitochondrial ATP production depends on OXPHOS complexes I to V located in the cristae in the inner mitochondrial membrane. As the surface area of cristae positively correlates with the amount of ATP produced by oxidative phosphorylation [71], we wondered whether cerium oxide nanoparticles would influence mitochondrial cristae. Indeed, the number of cristae was significantly lowered after 4h of CNP treatment (Fig 6). The loss of total ATP was observed after 24 and 48 hours (Fig 5), so we propose that an earlier loss in cristae number (after 4h of CNP, Fig 6) may be responsible for the later observed drop in the ATP amount, which must be verified in future experiments. The fact that the cristae number is recovering slowly after 24 hours (Fig 6) might indicate that the cells try to recuperate ATP production, but as CNP toxicity is constantly rising over time, the fate of the cells might already be determined at the 48 h timepoint.

In conclusion, a nanoceria mediated increase in mitochondrial fragmentation (Fig 4C) and a decrease of total ATP (Fig 5) following a loss of cristae (Fig 6) was observed in this study. We may speculate that it causes the cells to undergo apoptosis probably because of mitochondrial dysfunction and insufficient mitochondrial quality control. Quality control of mitochondria is crucial for the cell, because the loss of mitochondrial function is associated with various diseases, e.g. heart failure [80] or Parkinson´s disease [81]. However, in our melanoma cell model a nanoceria triggered and H_2_O_2_ mediated mitochondrial dysfunction is desired to force the tumor cells to undergo apoptosis. These data are in line with earlier published data postulating apoptotic cell death via a failure of mitochondrial quality control [82]. In addition, our data also match with other studies in which a depolarized mitochondrial membrane was observed in human colon cancer cells upon treatment with nanoceria [83]. Interestingly, a loss in mitochondrial membrane potential has also been found after MnSOD overexpression (and a subsequent rise in H_2_O_2_ generation) in MCF7 breast cancer cells [84].

**Fig 8:**
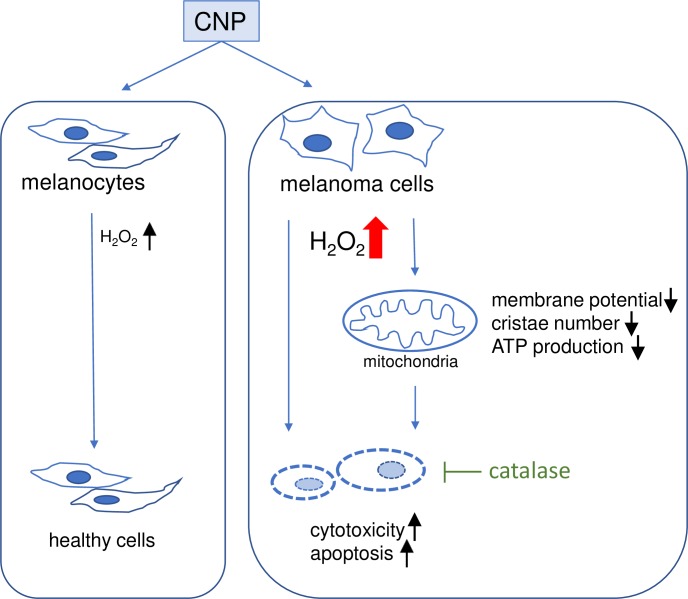
Selective effect of CNP on melanoma cells and melanoma mitochondria. It was shown in this study, that CNP exerts a selective cytotoxicity in A375 melanoma cells, accompanied by indices of mitochondrial dysfunction, i.e. loss of mitochondrial membrane potential, a reduced number of cristae and a decreasing amount of total ATP. The selective toxicity was abrogated by the enzyme catalase.

We show for the first time that in melanoma cells nanoceria directly affect i) mitochondrial fusion and fission by promoting mitochondrial fragmentation (Fig 4C), and ii) mitochondria cristae leading to a decrease of the number of cristae (Fig 6). Both fission and a lowered cristae number have also been reported as a result of hydrogen peroxide treatment [85]. The fact that preincubation with PEG-catalase fully rescued melanoma viability (Fig 7) further promotes our hypothesis that nanoceria increase the intracellular concentration of H_2_O_2_ exceeding a critical threshold and finally lead to changes in mitochondrial homeostasis with subsequent cell death. The tumor cells are no longer able to deal with the nanoceria mediated high ROS level, whereas normal (healthy) cells are not affected. In addition to the CNP/H_2_O_2_ mediated mitochondrial dysfunction it cannot be excluded so far that other H_2_O_2_ dependent factors may also play a role in the CNP/H_2_O_2_ initiated selective increase in cytotoxicity and apoptotic cell death of melanoma cells (Fig 8). This again underlines that nanoceria are a promising tool in cancer treatment, where still one of the most unwanted side effects is the toxicity of classical chemotherapeutics (e.g. anthracyclines) towards healthy cells or organs [86, 87].

## Supporting information

S1 Raw images (PDF)
