# Supplementary material for: CNP mediated selective toxicity on melanoma cells is accompanied by mitochondrial dysfunction
Source: PLoS One. 2020 Jan 17;15(1):e0227926. doi: 10.1371/journal.pone.0227926 (PMC6968876; doi:10.1371/journal.pone.0227926)
Supplement: S1 Raw images — (PDF) [file pone.0227926.s001.pdf]

Manuscript ID: PONE-D-19-25424R1 (revised)
S1_raw_images

The following images correspond to Fig3C of the manuscript

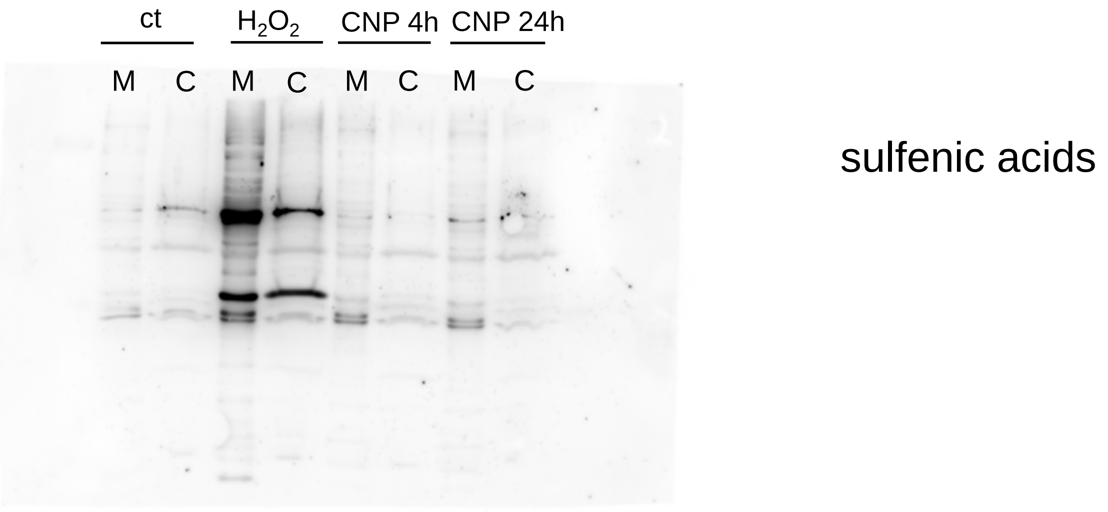

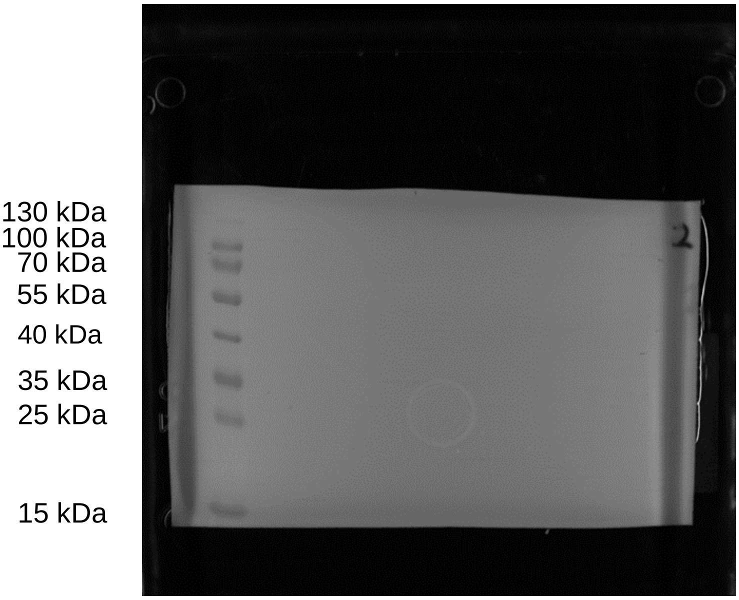

Upper panel:

A375 cells were separated into cytosolic (C) and mitochondrial (M) fraction after mock-treatment, incubation with H_2O_2 or incubation with CNP. Western Blot analysis was performed using an α-hapten antibody raised against the oxidation product of sulfenic acid and dimedone. The antigen-antibody complexes were visualized by an enhanced chemiluminescence system with the Fusion FX (Vilber Lourmat, Eberhardzell, Germany).

The image was taken at 8-14-18; exposure time was 3min 42sec.

Lower panel:

Corresponding PVDF membrane with markerMarker: PageRuler™Prestained Protein Ladder 10 to 180 kDa, Thermo Scientific

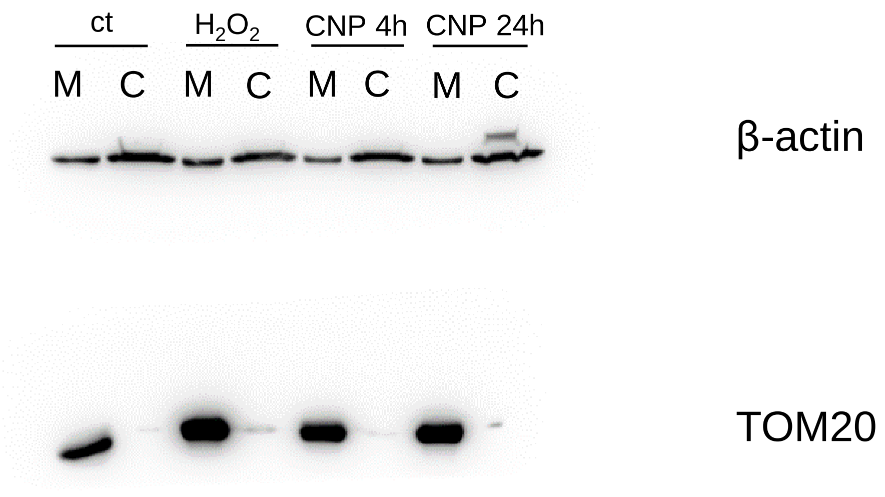

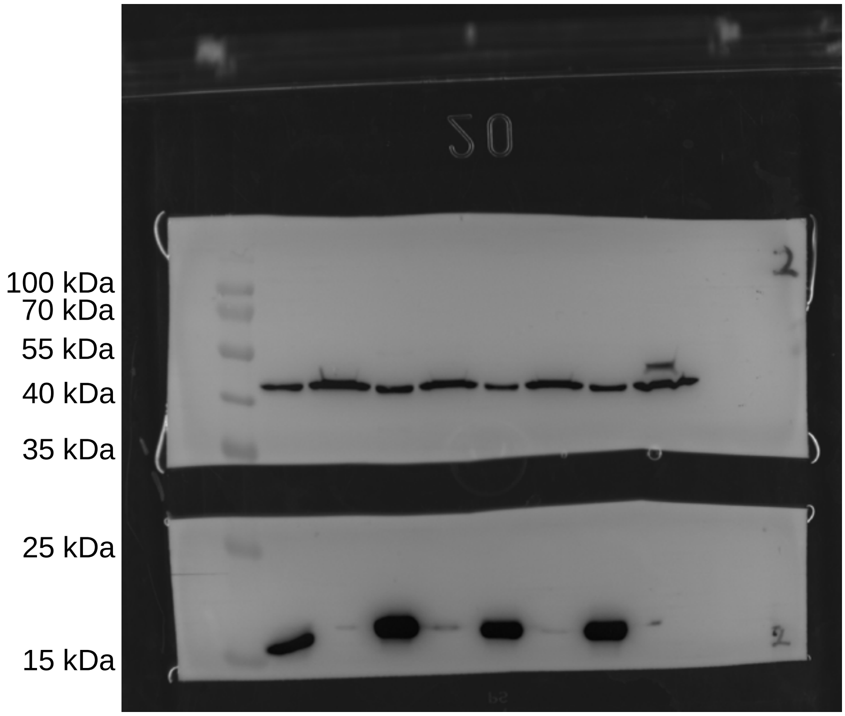

Upper panel:

A375 cells were separated into cytosolic (C) and mitochondrial (M) fraction after mock-treatment, incubation with H_2O_2 or incubation with CNP. Western Blot analysis was performed using anti-beta-actin and anti TOM-20 antibodies. Antigen-antibody complexes were visualized by an enhanced chemiluminescence system with the Fusion FX (Vilber Lourmat, Eberhardzell, Germany). The image was taken at 8-14-18, exposure time was 1min 72sec.

Lower panel:

Corresponding PVDF membranes with marker, merged with Western Blot

Marker: PageRuler™Prestained Protein Ladder 10 to 180 kDa, Thermo Scientific

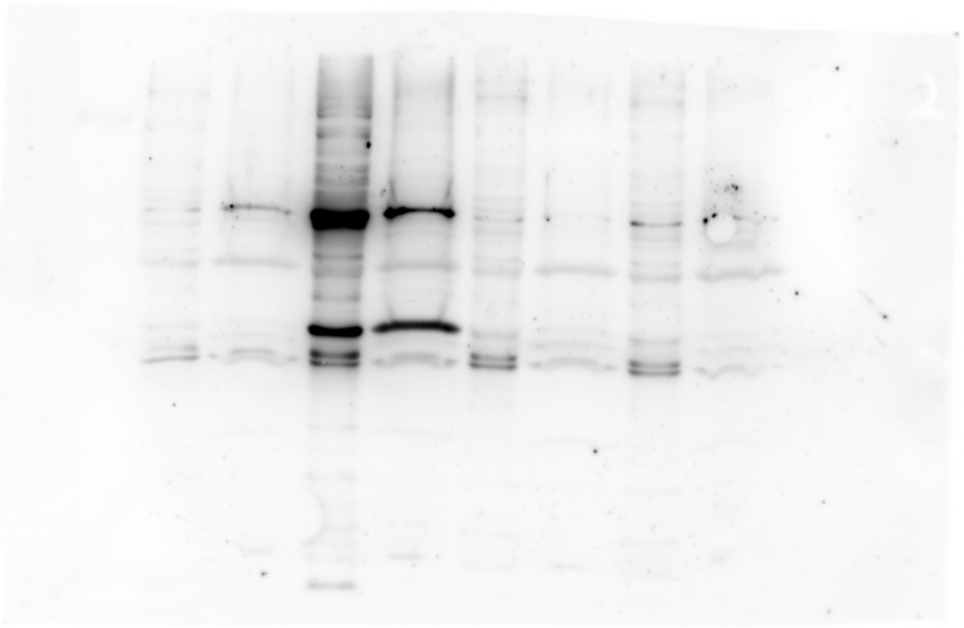
2

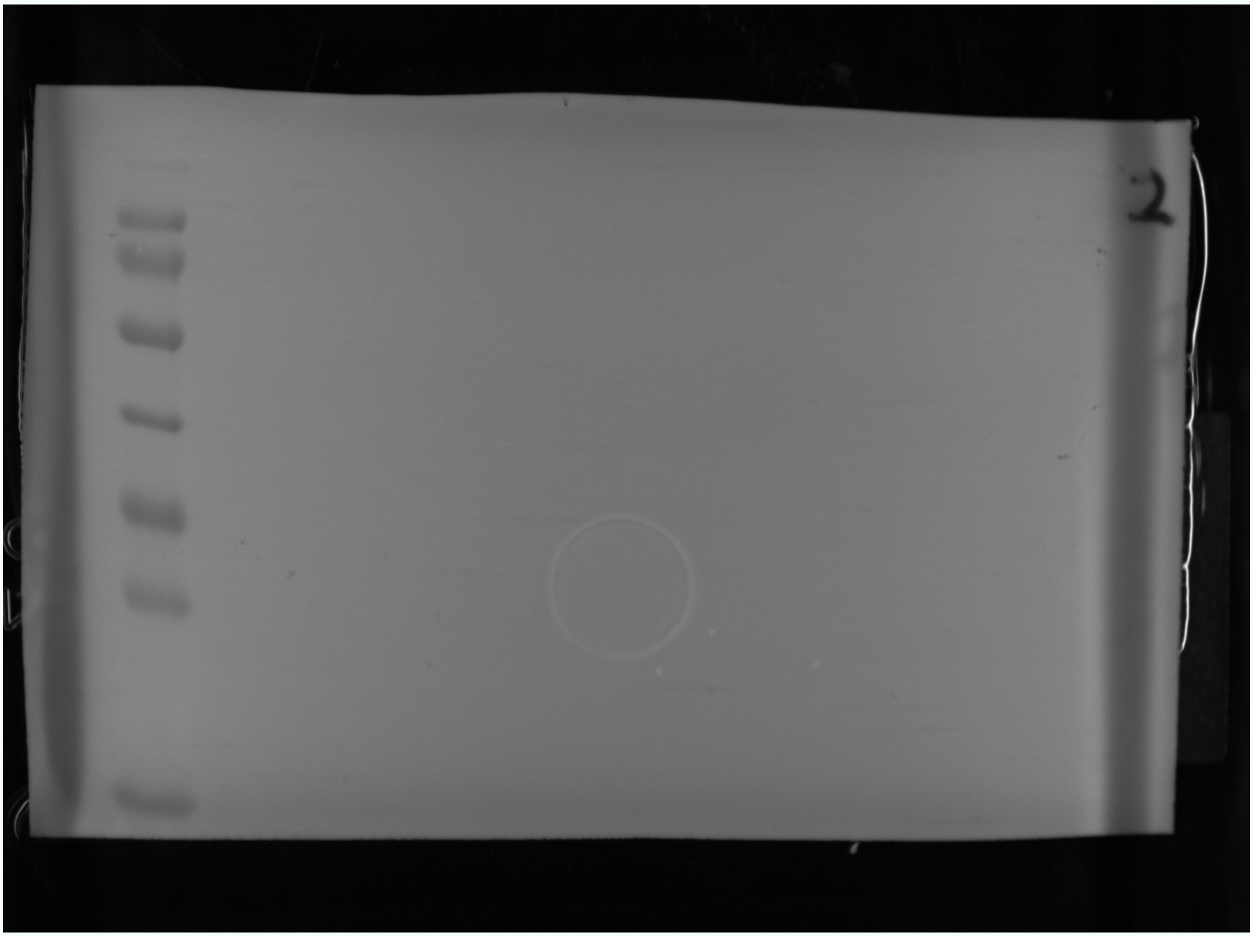
2

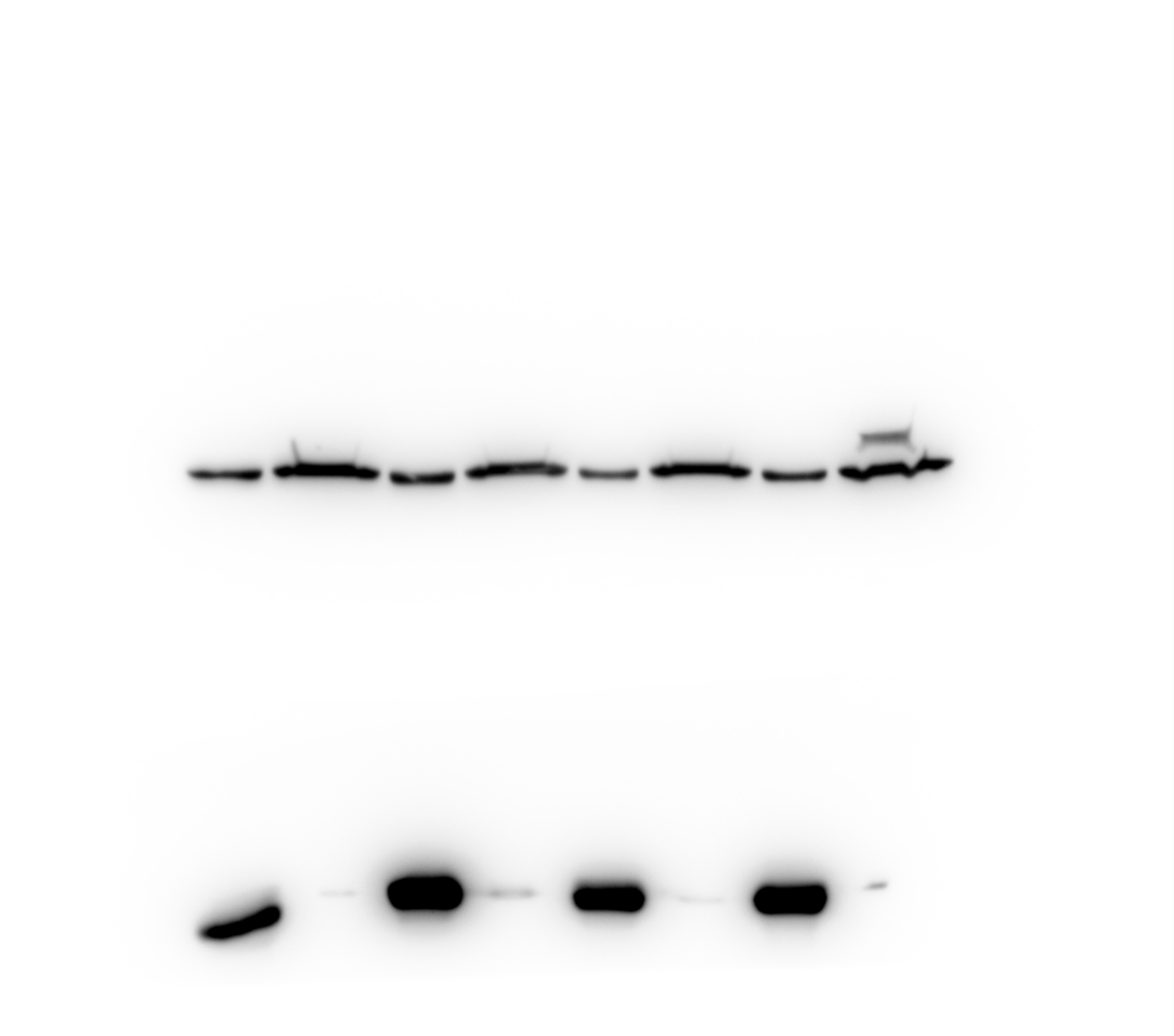

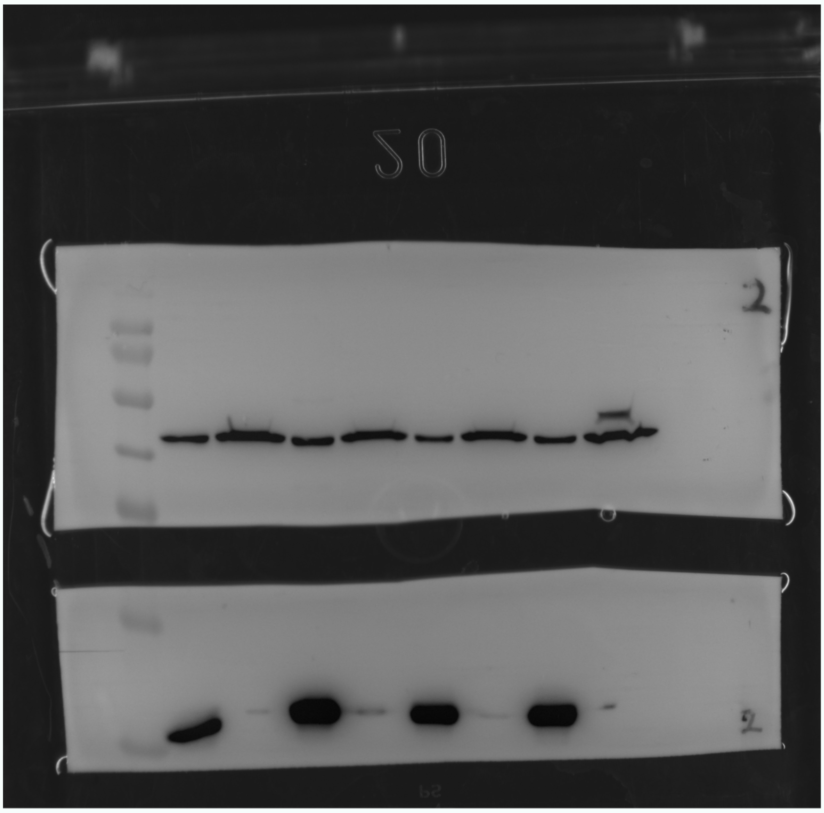
2
